# Bridging the gap: metabolic and endocrine care of patients during transition

**DOI:** 10.1530/EC-16-0028

**Published:** 2016-11-16

**Authors:** Anita Hokken-Koelega, Aart-Jan van der Lely, Berthold Hauffa, Gabriele Häusler, Gudmundur Johannsson, Mohamad Maghnie, Jesús Argente, Jean DeSchepper, Helena Gleeson, John W Gregory, Charlotte Höybye, Fahrettin Keleştimur, Anton Luger, Hermann L Müller, Sebastian Neggers, Vera Popovic-Brkic, Eleonora Porcu, Lars Sävendahl, Stephen Shalet, Bessie Spiliotis, Maithé Tauber

**Affiliations:** 1 Erasmus University Medical CentreRotterdam, The Netherlands; 2 University Children’s HospitalEssen, Germany; 3 Medical University and General Hospital of ViennaVienna, Austria; 4 Sahlgrenska University HospitalGöteborg, Sweden; 5 Istituto Giannina GasliniUniversity of Genova, Genova, Italy; 6 Hospital Infantil Universitario Niño JesúsMadrid, Spain; 7 University Hospital BrusselsBrussels, Belgium; 8 Queen Elizabeth HospitalBirmingham, UK; 9 Cardiff University School of MedicineCardiff, UK; 10 Department of Molecular Medicine and SurgeryKarolinska Institute and Department of Endocrinology, Metabolism and Diabetology, Karolinska University Hospital, Stockholm, Sweden; 11 Department of EndocrinologySchool of Medicine, Erciyes University, Kayseri, Turkey; 12 Department of PediatricsKlinikum Oldenburg, Medical Campus University Oldenburg, Oldenburg, Germany; 13 Belgrade University School of MedicineBelgrade, Serbia; 14 University of BolognaBologna, Italy; 15 Department of Women’s and Children’s HealthKarolinska Institutet, and Pediatric Endocrinology Unit, Karolinska University Hospital, Stockholm, Sweden; 16 The Christie HospitalManchester, UK; 17 University of Patras School of MedicinePatras, Greece; 18 Hôpital des EnfantsToulouse, France

**Keywords:** transition, developmentally appropriate healthcare, GH therapy, metabolic syndrome, quality of life

## Abstract

**Objective:**

Seamless transition of endocrine patients from the paediatric to adult setting is still suboptimal, especially in patients with complex disorders, i.e., small for gestational age, Turner or Prader–Willi syndromes; Childhood Cancer Survivors, and those with childhood-onset growth hormone deficiency.

**Methods:**

An expert panel meeting comprised of European paediatric and adult endocrinologists was convened to explore the current gaps in managing the healthcare of patients with endocrine diseases during transition from paediatric to adult care settings.

**Results:**

While a consensus was reached that a team approach is best, discussions revealed that a ‘one size fits all’ model for transition is largely unsuccessful in these patients. They need more tailored care during adolescence to prevent complications like failure to achieve target adult height, reduced bone mineral density, morbid obesity, metabolic perturbations (obesity and body composition), inappropriate/inadequate puberty, compromised fertility, diminished quality of life and failure to adapt to the demands of adult life. Sometimes it is difficult for young people to detach emotionally from their paediatric endocrinologist and/or the abrupt change from an environment of parental responsibility to one of autonomy. Discussions about impending transition and healthcare autonomy should begin in early adolescence and continue throughout young adulthood to ensure seamless continuum of care and optimal treatment outcomes.

**Conclusions:**

Even amongst a group of healthcare professionals with a great interest in improving transition services for patients with endocrine diseases, there is still much work to be done to improve the quality of healthcare for transition patients.

## Introduction

Monumental changes in human development occur during adolescence. Teens undergo a period of physical growth and sexual maturation, and pronounced cognitive development, and are eagerly forming their own identities to achieve independence from parents, establish adult relationships apart from their families, and find a vocation. It is a time that can be difficult to navigate for healthy children, but for adolescents with chronic health issues, the challenges can be exacerbated. At a time when patients want most to fit in with their peers, endocrine disorders that are not optimally managed may render patients with short stature, stunted puberty and sexual maturation, morbid obesity, and metabolic abnormalities.

Paediatric and adult endocrinologists and nurse specialists were invited to attend an expert meeting in Vienna, Austria, on 5–6 June 2015 entitled ‘Bridging the Gap: Metabolic and Endocrine Care of Patients during Transition’ to discuss and share how transitional care is being managed within various institutions (i.e., personal referrals vs informal clinics vs formal transition/adolescent centres) and to identify factors that are most critical for effective transition to occur without interruption of the patient’s continuum of care (e.g., financial and reimbursement issues; hospital administration support; and needs for patient support groups, more scientific data, and/or colleagues dedicated to transitional care). The care for patients with childhood-onset GH deficiency (CO-GHD), those born small for gestational age (SGA), girls with Turner syndrome (TS), patients with Prader–Willi syndrome (PWS), and survivors of cancers treated during childhood was addressed and, specifically, the role for GH therapy in patients with CO-GHD. Small-group breakout sessions also permitted discovery and discussion of uncovered perceptions about the role of paediatric vs adult endocrinologists in managing transition patients.

## Materials and methods

**Figure 1:**
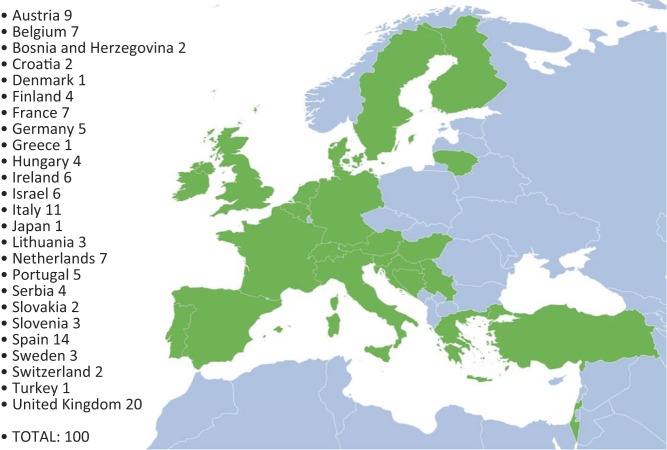
Numbers and geographical locations of delegates.

**Figure 2:**
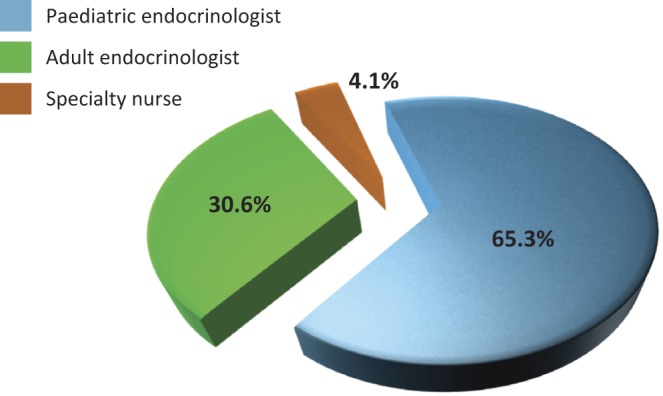
Delegate breakdown by specialty.

A total of 130 delegates representing 25 different countries attended the meeting (Fig. 1) with an approximate 2:1 ratio of paediatric to adult endocrinologists and approximately 4% nurse specialists (Fig. 2). The Scientific Planning Committee (SPC) was comprised of six experts in the field of endocrinology, who provided scientific and planning oversight for the overall programme and content. In addition, 13 faculty members presented at the meeting and 4 delegates moderated the breakout workshops.

The meeting was divided into three scientific sessions, grouped by content. Session I centred around the use of GH therapy during transition and beyond, including who, why and how to treat patients with GH. Session II was focused on why and how to follow-up and treat adolescent patients with TS and PWS. And in the last session, discussions dealt with the management of resulting endocrinopathies following cancer treatment during childhood. In addition, there were three breakout workshops designed to facilitate interactive discussions. Delegates were randomly separated into groups, each with a moderator, and given specific questions relating to the previous session’s topics to debate. A delegate from each workshop was selected by their peers to present a summary of their answers to the other attendees upon reconvening in the general session. Throughout the meeting, questions were asked of the delegates and their answers were recorded, analysed and projected to the audience by use of an Audience Response System.

## Results

Dr Allan Colver (UK), a paediatric specialist on the transition of adolescents with complex health needs from child to adult services, kicked off the meeting by highlighting the monumental developmental changes that occur in the adolescent brain during ages 11–25 years. He explained how these changes may impact the continuum of healthcare at a critical time when these young patients are seeking independence from their parents and beginning to navigate adult relationships and vocations (1). Understanding these complexities is key for achieving the following aspirations of transition, ‘the optimal goal is to provide care that is uninterrupted, coordinated, developmentally appropriate, psychosocially sound and comprehensive’ (2). Key elements to enhancing adolescent healthcare include conducting consultations with patients apart from their parents and an emphasis on physician–patient confidentiality so that teens feel comfortable discussing topics like drug use and misuse, and sexual health. In addition, flexibility with methods of communication including expanded clinic hours to accommodate school and college schedules and willingness to use non-traditional contact methods (i.e., texting, email messaging and phone/video calls) may enhance adolescent engagement. Using transition models in other chronic diseases such as diabetes, juvenile arthritis and cystic fibrosis, Dr Helena Gleeson (UK) provided a ‘road map’ of key initiatives that have proven to be effective when transitioning young people to adult care (Table 1).

**Table 1:** Critical steps in transition.

**Steps**	**Actions**	**Ref.**
1. Ask young people and their parents	• Inquire about patients’ feelings and concerns • Understand patients’ needs to balance independence with continued care and education • Solicit desired outcomes after transfer • Discuss transfer specifics (early preparation and clinic location)	(3)
2. Engage/train colleagues	• Different perceptions of levels of competency and involvement of paediatric vs adult physicians during transition care • Lack of training and inadequate resources are main barriers to successful transition service	(4)
3. Choose a clinic structure	• Multidisciplinary collaboration across all health sectors, including primary and secondary care, enhances successful transfer of care	(5)
4. Structured transition programme	• Requested by adolescents with discussions starting early in disease course, possibly soon after diagnosis • Example of successful generic transition programme is ‘Ready Steady Go’, implemented within a large National Health Service teaching hospital in the UK	(3) (6) (7)
5. Help with navigation	• Both hands-on and hands-off approach beneficial; a transitional care coordinator can assist with young people navigating transition and transfer and access to adult services	(8)
6. Monitoring	• Useful tools to monitor successful outcomes can include evaluation of specific disease markers (e.g., HbA1c), quality-of-life questionnaires, monitoring of attendance/adherence at clinic visits and reduced morbidity/mortality	(9)
7. Engaging the disengaged	• Get adolescents involved in monitoring and design; use technology-based transition interventions designed for adolescents with diverse chronic illnesses	(10)

**Figure 3:**
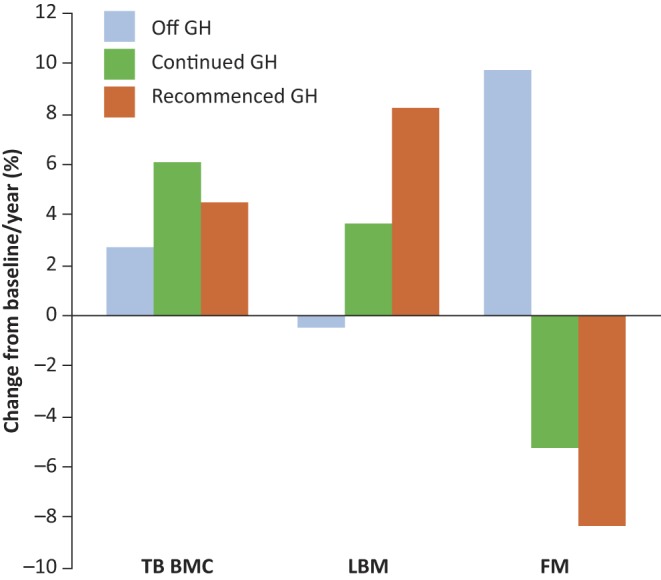
Effects of discontinuation, continuation, and recommencement of GH replacement therapy on bone mineral density and body composition in the transition period (12). FM, fat mass; LBM, lean body mass; TB BMC, total body bone mineral content.

Both adult and paediatric endocrinologists are faced with similar uncertainties when managing the care of transition patients with GH abnormalities. They must consider which patients to test and what test to use for determining continued GH deficiency (GHD) and then decide which patients could benefit from continued treatment, and whether continued treatment is safe. Data presented in the first scientific session largely support the role of GH therapy during the period of transition and beyond. Patients with childhood-onset hypopituitarism, who have severe GHD and low insulin-like growth factor-I (IGF-I) levels, are likely to have permanent GHD. Testing should be undertaken after at least one month after discontinuation of GH therapy and patients with discordant tests or those at risk of permanent GHD should be carefully followed for either progressive deterioration or potential recovery of pituitary function over time (11, 12). Following attainment of adult height, additional benefits of GH therapy include improved body composition and bone mineral density (Fig. 3), as well as improved lipid profiles and improved quality of life (QoL) measures. A delay in continuing GH therapy in young adults with CO-GHD appears to negatively impact many of these effects. One major issue that has been the focus of numerous studies and in-depth discussions amongst the various endocrine societies is the safety of continued GH therapy (13, 14). Dr Lars Sävendahl presented a critical and in-depth overview of recently published papers on long-term safety evaluations based on analyses of various European registries participating in the Safety and Appropriateness of GH treatments in Europe (SAGhE) consortium (i.e., Belgium, France, Germany, Italy, the Netherlands, Sweden, Switzerland and the United Kingdom). The session concluded that while awaiting the final results of the whole SAGhE consortium, patients may continue GH treatment safely, a position that is consistent with statements released by the various societies including the European Society for Paediatric Endocrinology (ESPE), the Endocrine Society (ENDO), and the Pediatric Endocrine Society (PES). During the first workshop, each breakout group was asked a series of questions about the topics discussed in Session I to get a better understanding of actual clinical practices. The majority of delegates believed that it was the responsibility of the paediatric endocrinologist to perform the re-testing of continuing GHD into adulthood. However, some delegates thought that since the adult endocrinologist would be making the decision whether or not to continue GH therapy in adulthood, they should be the ones to conduct the re-testing. In terms of which patients to test, responses varied. Some delegates recommended always testing patients with partial or severe GHD, while others recommended stopping GH therapy for one month and then re-testing all patients whose IGF-I levels were abnormal (and not attributable to other concurrent organic or genetic disease). If physicians were torn between deciding whether or not to re-rest, delegates recommended erring on the side of safety and performing GH stimulation testing. Delegates used the insulin tolerance test, arginine, glucagon, or arginine–GH releasing hormone stimulation testing. They stressed that testing physicians use the test that they are most comfortable administering and interpreting. Many delegates reported a lack of formal transitional centres, but relied, instead, on informal discussions with colleagues or personal referrals when transitioning from paediatric to adult care. When deciding how GH therapy should be managed and which parameters should be monitored, there was near-unanimous agreement that IGF-I levels should be evaluated, but delegates also stressed the importance of monitoring bone mineral density (BMD) and lipid profiles, and some physicians also assess fasting glucose/glycated haemoglobin levels (HbA1c) and QoL measures.

The second scientific session of the meeting explored transitional care of patients with TS and PWS from the perspectives of both paediatric and adult endocrinologists and how to manage children at risk for metabolic syndrome beyond adolescence. Some girls with TS are plagued by decreased adult height, gonadal dysgenesis, endocrine disturbances, cardiovascular abnormalities and psychosocial problems. Successful navigation from paediatric to adult care is particularly difficult in these patients who need life-long follow-up and screening. Comprehensive summaries of issues on medical care in patients with TS have been published that provide helpful checklists for use in clinical practice (16, 17, 18, 19). One of the main goals during adolescence is to mimic physiological puberty, so the current recommendation is to begin oestrogen therapy around the ages of 11–12 years in the presence of suspected ovarian failure. Additional data are needed to determine the optimal dosage or whether starting oestrogen therapy earlier would convey additional benefit. Growth hormone therapy, at supraphysiological doses, can improve adult height (gain of approximately 8 cm) with early treatment, preventing short stature in the majority of patients. Improvement in adult height is superior when administered earlier rather than later in the course of the condition (20). Patients should be screened for cardiovascular abnormalities with mandatory regular follow-up visits. Hormone replacement therapy in patients with TS improves BMD, blood pressure and liver enzymes with no set ‘window of opportunity’ for administration (13, 14, 15, 16, 17, 18, 19, 20, 21, 22, 23). Screening of adolescent and adult patients should include cardiac evaluations (blood pressure, auscultation, echocardiography and MRI); thyroid status; metabolic health (body mass index, level of physical exercise and diet, blood sugar and lipid profile levels, and liver enzymes); BMD (osteoporosis surveillance); hearing; and urinary testing. Both paediatric and adult physicians agree that treatment clinics should be multidisciplinary, but anchored in one department. What remains unclear is how best to implement the recommended care in adult women with TS, many of whom are lost during the transitional period.

Patients with PWS share many similarities with patients diagnosed with GHD. Patients with PWS present with a host of disorders (i.e., multiple hormone deficiencies, hyperphagia, obesity, sleep apnoea, intellectual disabilities, and behavioural and psychiatric issues), many of which appear, or worsen, during adolescence. Optimal healthcare management during this critical time requires a multidisciplinary transition team. Dr Maithé Tauber, director of the Reference Centre for PWS in France, includes both the paediatrician and the adult endocrinologist during the entire transition process. Through a minimum of three visits, two in the paediatric setting and one visit in the adult setting with both paediatric and adult physicians attending, patients are transitioned to adult care (Table 2).

**Table 2:** Example of successful steps in transition of patients with PWS.

**Visit**	**Clinic setting**	**Actions**
1	Paediatric	• Paediatrician assesses adult height and patient undergoes multidisciplinary evaluation (i.e., rheumatologist, cardiologist, psychologist, etc.) • Adult endocrinologist receives oral summary of the patient’s medical history and detailed description of the present situation (including medical examination, socio-educational aspects of the situation, psychological and relational issues, medical therapies, family structure, etc.) • Patients are given opportunity to talk with physicians alone, without parents, to voice concerns about sexual and reproductive issues, psychological difficulties, and autonomy in everyday life
2	Paediatric	• Can comprise multiple visits • Visits occur every 6 months and include complete medical check-up • Through joint decision by physicians, patient, and their family, date is set for first visit in adult endocrinology unit; could be as soon as 6 months or up to 2 years later, depending on complexity of the situation and patient’s readiness for transition • Whenever patient is ready for first ‘adult’ visit, paediatrician sends complete medical report to adult clinic and sets appointment
3	Adult	• Evaluation performed during last transition visit is similar to the other check-ups undertaken in paediatric unit; primary difference is environmental: s/he is now considered an adult and begins management under care of adult specialists

Several studies report an insufficient rise in GH upon stimulation (8–67%) and low IGF-I levels in the majority of adult patients (75–91%) with PWS indicating reduced activity of the GH/IGF-I axis (24, 25, 26). There are conflicting results regarding GHD in adult patients with PWS with more recent papers reporting fewer cases of GHD, perhaps due in part to fewer morbidly obese patients. While hormone replacement therapy does not change the intrinsic abnormalities of PWS, treatment with GH improves body composition, physical fitness, bone size and strength, and QoL (27). Clinical experience suggests a positive response with replacement of sex steroids, confirmed in men with PWS in the only treatment trial conducted to date (28). Strict diet and regular physical exercise also play key roles in overall patient health. Early diagnosis, combined with multidisciplinary care including GH replacement and use of other hormonal therapies, can ease PWS complications, ameliorate comorbidities, and improve QoL and transition to adult life in these patients (29). However, additional studies with hormone replacement, particularly during transition, are needed.

The role of GH therapy in children born small for gestational age (SGA) has been well documented to be effective at increasing linear growth and improving body composition. Growth hormone therapy in these children can also induce higher insulin levels which led to speculation that these patients may be at increased risk for development of metabolic syndrome. However, studies have shown that in children born SGA, GH-induced insulin insensitivity disappeared after discontinuation of GH, at least after 6 years of discontinuation of GH therapy (30). In addition, the beneficial effects of GH therapy on systolic and diastolic blood pressure indices and serum lipids remained unchanged even after 6 years of discontinuation of GH therapy (31). Children are followed up regularly while receiving GH therapy; however, beyond adolescence, knowledge is limited as to how often to monitor these patients. Long-term follow-up data are required to make recommendations during transition and adulthood, but it is imperative to remind SGA patients not to become overweight or obese and to seek low-threshold clinical care in the presence of fatigue, weight gain, and a family history of diabetes, cardiovascular disease or hypertension.

The goals of the second breakout workshop were to garner information on current clinical practices with regard to treatment and follow-up of patients with TS and PWS. The majority of delegates reported that in their centres/hospitals, adult endocrinologists lead the management of patients with TS when they emerge from paediatric endocrine care, but with input from a multidisciplinary team including gynaecologists, cardiologists, general practitioners and psychologists. While published guidelines on the management of TS patients exist, treating physicians must stay abreast of the most recent publications to ensure adherence. Fertility in these patients is an important issue and the consensus was to begin discussions with patients beginning around ages 11–12 years, depending on their maturity level, using simple terms. Additional specialists, i.e., obstetricians/gynaecologists, geneticists, etc., may be included in these conversations to provide robust information. Hormone replacement therapy should be individualised, but in most patients, treatment should be started at low doses and increased slowly over a 2-year time period to mimic physiological puberty. Many delegates prefer the use of transdermal patches and then switch to oral medications as the patient matures.

Decisions about whether to continue GH therapy after adult height attainment in adolescents and adults and how best to evaluate the hypothalamic/pituitary/gonadal axis in patients with PWS were not so straightforward. While the majority of responders assume that continuing GH therapy may result in increased adult height and improved lean body mass, exercise capacity, QoL and BMD, some are concerned about the effects of GH on insulin resistance and diabetes in adolescent and adult with PWS. With a lack of substantive data and the fact that GH therapy is non-reimbursable in many countries after attainment of adult height, delegates suggested monitoring IGF-I levels, especially in patients with confirmed GHD at transition, evaluating gonadotropin levels, and watching for early signs of obesity development, increasing hypotonia, decreased QoL, and a low activity level.

The third and last scientific session of the meeting focused on patients with endocrine sequelae as a result of craniopharyngiomas or cancer therapy administered during childhood. Dr Hermann Müller opened the session with a discussion of the challenges in the treatment of patients with craniopharyngiomas (CPs). Since childhood-onset CPs often affect the hypothalamic and pituitary regions as well as the optic chiasm, regardless of the degree of resection, patients frequently experience relapse and progression events during long-term follow-up. For these reasons, treatment of CP should be viewed as chronic (32). During initial diagnosis, experienced multidisciplinary teams should formulate treatment plans for postoperative follow-up and rehabilitation. Gross total resections should be avoided in cases of hypothalamic involvement in an effort to spare any further damage to the sensitive region (32). And since endocrinopathies are relevant causes of death in patients with CPs (33, 34), it is extremely important for patient health to be guided and monitored by an endocrinologist.

The last group of patients discussed in this meeting were survivors of childhood cancers and how to treat and manage endocrine sequelae during transition and into young adulthood. While advances in anticancer treatments have greatly increased survival in children, the damage of these therapies to organ systems may not be evident for many years (35). Data from the Childhood Cancer Survivors Study, a large, multi-centred, retrospective study comprised of adults who have survived for at least 5 years post cancer therapy, have shown a high risk for chronic health conditions. The rates are especially high for endocrinopathies, i.e., premature gonadal failure, thyroid disease, osteoporosis, and hypothalamic and pituitary dysfunction (35). Of paramount importance to many adolescents and their families is the implication of neoplastic therapy on future fertility. While cryopreservation of sperm is widely used in males undergoing oncologic therapy, options for preservation of fertility in female patients are more complex (36). Treating physicians should implement discussions early on so that families can determine the best options for fertility preservation. It is important to remember that obesity and gonadal function are intrinsically linked and perturbations of either can adversely affect the other (37, 38). The risk of developing metabolic syndrome is also high in Childhood Cancer Survivors (CCS), particularly in patients who have received radiation therapy. Patients who have received cranial radiotherapy (CRT) are at higher risk for anterior hypopituitarism (39). A report from the St Jude Lifetime Cohort study found that in 748 CCS treated with CRT, 348 had an IGF-I standard deviation score (SDS) <−2 indicating a prevalence of severe GHD in nearly 50%. And of those severe GHD patients, only one was receiving GH therapy. Untreated GHD results in significantly decreased muscle mass and exercise tolerance, increased abdominal obesity, low energy expenditure, muscle weakness, and impaired QoL. In this study, the impairment was related primarily to the GHD rather than the original cancer diagnosis and treatment. Reassessment of GH status should be mandatory after achievement of adult height in all patients who received GH therapy during childhood for radiotherapy-induced GHD. While this strategy should be effective at identifying many of the patients with severe GHD, guidelines still need to be developed for repeated retesting of GH status in patients with mild-to-moderate GHD at adult height.

During the last breakout workshop, delegates discussed and debated follow-up and treatment in CCS. While everyone agreed that these patients should have long-term follow-up by an adult endocrinologist, there was debate about the frequency of the visits. Timing can depend on the type of cancer and antineoplastic treatments administered and, in some countries, the frequency may be dictated by healthcare coverage. During the follow-up visits, routine tests should include a physical examination, anterior pituitary hormone function, thyroid hormone assessment, metabolism (BMI, lipid profile, glycated haemoglobin, glucose levels, etc.), assessments of QoL, bone mineral density, and fertility parameters. Additional tests may be necessary based on type of cancer and therapies administered, e.g., assessment of cardiac function in patients who received cardiotoxic regimens. GH replacement in patients found to be GH-deficient is generally considered safe with follow-up similar to other adults with GHD (13). When transitioning from a paediatric endocrinologist to adult internist–endocrinologist, it is also important that the specialists work together as a team to ensure that adult providers have a clear and detailed medical history including a summary of previous treatments so as to anticipate/plan for future complications. It is also beneficial if the paediatric oncologists begin discussions with their patients about fertility, stress the importance of maintaining a healthy weight and balanced diet, and introduce the concept that survival often comes with a price (a lifelong commitment to carefully monitoring their health with the expectation that side effects of their disease and/or treatments are likely). Ideally, members of the transition team would include paediatric oncologists and radiotherapists who understand the long-term consequences of antineoplastic therapies; psychologists who can help patients deal emotionally with very complex diseases; paediatric and adult endocrinologists who can provide follow-up and navigate hormonal issues; reproductive health specialists, nurse specialists to aid in coordinating care, dieticians to help patients manage their weight and diet; and social workers to provide assistance with reimbursement and employment.

Guidelines for transitional care for adolescents with endocrine and metabolic disorders can be modelled after transitional care programs for other chronic diseases. Successful programs have included courses for adolescents on self-management education, skills training, joint provider sessions involving members from the both paediatric and adult treatment teams, use of a transition coordinator, and utilisation of specialised young adult clinics (40, 41, 42, 43). Non-institutional barriers to successful transition can include a host of perceptions about the roles and responsibilities of the adult vs the paediatric endocrinologists, beliefs held by the parents, and expectations of the patients (Table 3).

**Table 3:** Hurdles and solutions in achieving cohesive and seamless transitional care.

**Transition team member**	**Possible viewpoints**	**Possible solutions**
Paediatric endocrinologist	Might think that: • Adult endocrinologists are not cognisant of the complexity of the problem • Adult endocrinologists are not willing to provide adequate time for visits/discussion	• All providers need to work together to craft developmentally appropriate healthcare so young patients become more knowledgeable and responsible for managing their conditions • Emphasise physician–patient confidentiality so that teens feel comfortable discussing topics like drug use, misuse, and sexual health
Adult endocrinologist	Might think that: • Paediatric endocrinologists are overprotective and do not sufficiently prepare paediatric patient for move to adult care	• All providers must agree to sharing information and recognising that some overlap is necessary for a seamless conversion • Willing to be flexible with methods of communication with transition patients, e.g., expanded clinic hours to accommodate school/college schedules; use of non-traditional contact methods (email, text)
Parents	• Parents may be unwilling to readily trust a new provider, especially if the adult endocrinologist has not been involved in transitional plan • Parental trust issues can influence the young person	• Include parents in transition process to witness growth of relationship between child and provider and ease concerns about turning over the primary responsibility of healthcare management to patient
Young people	• Young people have become accustomed to allowing parents to manage healthcare consideration • Young people may feel that paediatric endocrinologists have known them and their medical condition for many years and are, therefore, more knowledgeable than the new adult endocrinology provider	• Young people need to become more involved in making healthcare decisions so that they become more adept at managing their disease

While many paediatric providers and parents feel that delaying transition until after completion at university permits continuity of care, post-transition surveys from young adults indicated that young people felt more independent and in charge of their disease during this time and that their new adult providers were more supportive of their decision-making and autonomy (44). While transition should begin in early adolescence (11–13 years of age) at the latest, deciding ‘when’ to transfer should depend largely on the readiness of the young person including their preferences, their level of independence in healthcare, and various condition factors. In addition, it would be beneficial to establish a relationship with an adult provider before issues of adulthood (e.g., stress of hospitalisation, pregnancy, etc.) force a meeting in less than favourable circumstances.

Throughout this specialty meeting, delegates have shared their experiences and ideas about transitional healthcare primarily in the background of various endocrinopathies. However, there are a number of factors for providers to consider incorporating into whatever model of transitional care they are using, regardless of patient diagnosis (Table 4).

**Table 4:** Factors contributing to successful transitional healthcare.

**Disease-specific considerations** (GHD, PWS, TS, SGA, CCS)	**Generic considerations**
• Identify which patients to test for endocrine dysfunction and which would benefit from GH replacement • Decide which stimulation test to use • Use specific disease markers to gauge successful intervention, i.e., IGF-I levels, BMD, lipid profiles, and possibly HbA1c and QoL measures • Aim to improve adult height, normalise body composition, and bone density • Preserve fertility (particularly for patients with TS or CCS) • Determine timing of subsequent follow-up visits once GH therapy is stopped	• Be approachable and welcoming; adolescents like eye contact, facial expressions, friendliness • Make the clinic environment more youth friendly, i.e., relaxed dress code for providers, availability of magazines/television channels geared towards young adults • Permit consultations with adolescents alone, clearly emphasise confidentiality, and tell patients what to expect in age-appropriate terms • Be willing to hear concerns about sexual health, substance abuse and mental health issues • Institute flexible access, i.e., extended clinic hours to accommodate school/job schedules, and use of email/texting/video chats • Avoid having young adults make key decisions at times of excitement or stress (i.e., holidays, final examinations, etc.) • Remember that healthcare is just one of many concerns for the young patient as they are also dealing with education, budding sexuality, separation from parents, navigating friendships, etc. • Engage young adults in self-management education and skills training • Work to establish joint provider sessions involving members from paediatric and adult treatment teams • Utilise a transition coordinator and/or specialised young adult clinics

BMD, bone mineral density; CCS, childhood cancer survivors; GHD, GH deficiency; IGF-I, insulin-like growth factor I; PWS, Prader–Willi syndrome; QoL, quality of life; SGA, small for gestational age; TS, Turner syndrome.

## Discussion

We have learned that even amongst a group of healthcare professionals who have an interest in improving transition services for patients with endocrine diseases, there is still much work to be done to improve the quality of healthcare for patients moving from paediatric to adult care. While the principles of quality healthcare during transition are the same for all young people, regardless of endocrine condition, methods of transition (i.e., informal discussions, formal transition centres, etc.) can differ amongst young people. Paediatric and adult endocrinologists must be willing to work together, sharing information and recognising that some overlap is necessary for a seamless conversion. Adolescent patients need developmentally appropriate healthcare to become more knowledgeable and responsible for managing their disease. And parents need to be included in the transition process to witness the growth of the relationships between their child and his/her providers so that they develop a level of confidence in ultimately turning over the primary responsibility of healthcare management to the patient. Whether through development of a standardised referral system, informal discussions amongst colleagues, or establishment of formal transition centres, all gap-bridging efforts are welcome to ensure that the needs of adolescents with chronic endocrine conditions are being met.

## Declaration of interest

Jesús Argente: nothing to disclose. Jean DeSchepper: received fees for lectures from Pfizer, for research projects from Novo-Nordisk and Pfizer, and for national and international advisory boards from Ferring and Ipsen. Helena Gleeson: nothing to disclose. John W Gregory: received speaker fees for lectures at industry-sponsored meetings from Pfizer and Bayer and travel grants from Pfizer and Ipsen. Berthold Hauffa: served in advisory boards for Alexion, Ipsen, Merck Serono, Novo Nordisk and Pfizer; received honoraria for lectures and for the scientific organization of meetings from Bayer, Ferring, Hexal/Sandoz Ipsen, Jenapharm, Merck Serono, Novo Nordisk and Pfizer; and received research grants from Hexal/Sandoz and Pfizer. Gabriele Häusler: received research grants on topic-related projects via Medical University of Vienna (§27) and lecture fees from Ipsen, Pfizer and Novo Nordisk. Anita Hokken-Koelega: received independent research grants for multicentre studies from Pfizer, Novo Nordisk, Ipsen and Lilly; received lecture fees from Pfizer and Novo Nordisk; and received fee for KIGS Steering Committee participation. Charlotte Höybye: member of Nordinet IOS, Patro and Swedish KIMS board (Pfizer); investigator for Nordinet, Patro and SweGro studies; received lecture fees from Novo Nordisk, Sandoz and Pfizer. Gudmunder Johannsson: received lecture fees from Pfizer, NovoNordisk, Merck Serono, Shire and Otsuka and has been a consultant for Viropharma, Shire and AstraZeneca. Fahrettin Kelestimur: nothing to disclose. Anton Luger: received honoraria for presentations and/or consultations from Pfizer, Ipsen, Novo Nordisk, Merck Serono; research support for the Division of Endocrinology and Metabolism of the Medical University of Vienna with unrestricted grants from Pfizer, Ipsen and Novo Nordisk. Mohamad Maghnie: received research contracts/grants and consulting/lecture fees from Eli Lilly, Ipsen, Ferring, Merck Serono, Pfizer, and Novo Nordisk. Hermann Müller: nothing to disclose. Sebastian Neggers: received research grants and lecture fees from Ipsen and Pfizer. Vera Popovic-Brkic: served as a consultant speaker for Pfizer and Novartis. Eleonora Porcu: nothing to disclose. Lars Sävendahl: received lecture honoraria from Ferring, Ipsen, Merck Serono, Novo Nordisk, and Pfizer; recipient of GGI research award sponsored by Merck Serono; president of the NordiNet International Study Committee; and work package leader for the EU-SAGhE project. Stephen Shalet: received lecture fees from GH-producing companies and non-profit organisations. Bessie Spiliotis: nothing to disclose. Maithé Tauber: received research funding from pour la recherché from Pfizer, Novo Nordisk, Ipsen and Sandoz; conference participation fees from Novo Nordisk and Ipsen; scientific board meeting participation fees from Merck Serono, Ipsen and Novo Nordisk; and consultant for Alizé Pharma. Aart-Jan van der Lely: received consultancy and speakers fees from Pfizer and Novartis.

## Funding

Pfizer Ltd. initiated and sponsored the ‘Bridging the Gap: Metabolic and Endocrine Care of Patients during Transition’ meeting. SPC and Faculty members received honoraria from Pfizer Ltd. for their participation. Medical writing support for preparation of the manuscript was provided by Pfizer Ltd. Prof. Allan Colver (Newcastle University, UK) presented on the Specific Needs of the Adolescent with Chronic Disease during Transition but did not contribute to the manuscript preparation. Prof Colver had no conflict of interest.
